# Supplementary material for: Antioxidant Guided Fractionation of Blackberry Polyphenols Show Synergistic Role of Catechins and Ellagitannins
Source: Molecules. 2023 Feb 17;28(4):1933. doi: 10.3390/molecules28041933 (PMC9967577; doi:10.3390/molecules28041933)
Supplement: Supplementary file 1 [file molecules-28-01933-s001.zip › molecules-2210734-supplementary.pdf]

Article

Antioxidant guided fractionation of blackberry polyphenols show synergistic role of catechins and ellagitannins

Katerina Tzima[1], Gontorn Putsakum[1] and Dilip K. Rai[1*]

[1] Teagasc Food Research Centre, Ashtown, Dublin D15KN3K, Ireland
* Correspondence: dilip.rai@teagasc.ie ; Tel.: +353 (0)18059569

Citation: Lastname, F.; Lastname, F.; Lastname, F. Title. *Nutrients* **2023**, *28*, 1933. https://doi.org/10.3390/molecules28041933

Academic Editor: Firstname Lastname

Received: date
Accepted: date
Published: 17 February 2023

Copyright: © 2023 by the authors. Licensee MDPI, Basel, Switzerland. This article is an open access article distributed under the terms and conditions of the Creative Commons Attribution (CC BY) license (https://creativecommons.org/licenses/by/4.0/).

Supplementary Materials:

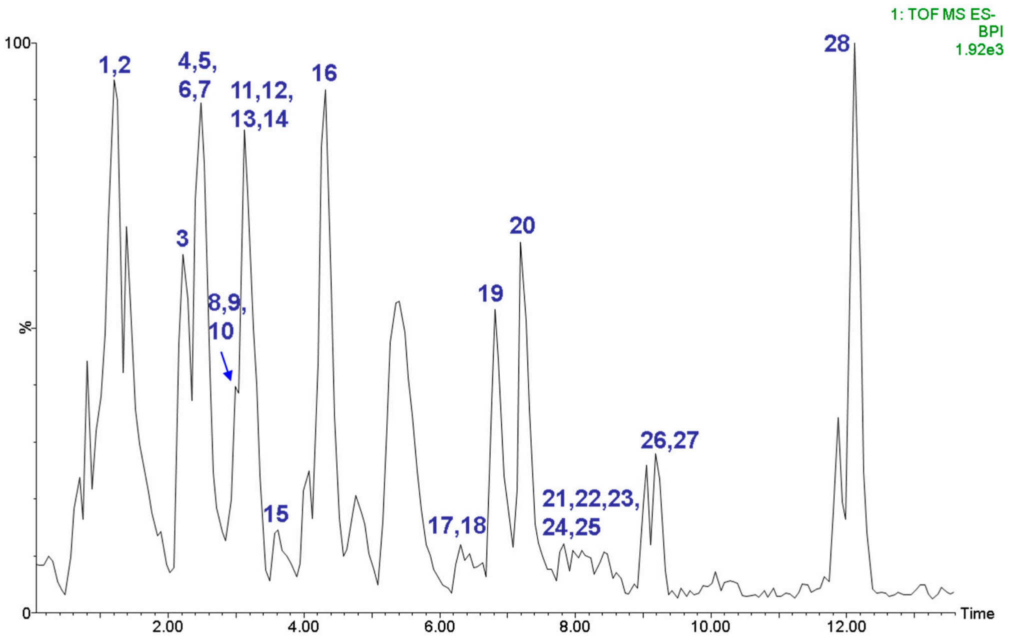

Figure S1. Representative base peak intensity (BPI) chromatogram of phenolic rich blackberry crude extract. Peak 1) Gallic acid, 2) Neochlorogenic acid, 3) Catechin, 4)Protocatechuic acid, 5) Dihydromyricetin rhamnoside, 6) Cyanidin-3-*O*-glucoside, 7) Cyanidin-3-*O*-rutinoside, 8) Procyanidin trimer C type, 9) 3-*p*-Coumaroylquinic acid, 10) Procyanthocyanidin type, 11) Cyanidin-3-*O*-sophoroside, 12) Procyanidin B2, 13) Cinnamtannin A2 14) 3-Feruloylquinic acid, 15) Procyanidin trimer C type, 16) Epicatechin, 17) Rutin, 18) Epigallocatechin, 19) Lambertianin C isomer, 20) Sanguiin H-6/Lambertianin A, 21) Ellagic acid pentoside, 22) Ellagic acid, 23) Quercetin-3-*O*-galactoside, 24) Quercetin-3-*O*-glucoside, 25) Quercetin-3-*O*-hexoside, 26) Quercetin-3-pentoside, 27) Kaempferol-3-*O*-rutinoside and 28) Quercetin.

Table S1. Retention time (t_R), MRM transitions, collision energy and cone voltage for the standards used and the phenolic compounds that were quantified in the different fractions of the phenolic-rich blackberry extract [1, 2].

Reference Compound	t_R (min.)	MRM Transition (*m*/*z*)	Cone Voltage (V)	Collision Energy (eV)
Protocatechuic acid	2.59	153.06 → 108.94	29	16
		→ 80.88		18
Cyanidin-3-*O*-glucoside	3.60	448.61 → 287.20	30	15
		→ 316.60		15
Cyanidin-3-*O*-rutinoside	3.70	594.92 → 286.80	30	20
		→ 449.20		20
Cyanidin-3,5-sophoside	3.72	611.16 → 285.10	55	24
		→ 475.20		35
Cinnamtannin A2	3.75	1155.12 → 577.32	40	40
		→ 984.45		40
Procyanidin B2	3.76	577.30 → 288.94	55	26
		→ 406.90		28
Epicatechin	4.08	289.24 → 244.95	47	14
		→ 202.74		16
Lambertianin C	4.45	1401.87 → 300.09	65	35
		→ 632.61		35
Sanguiin H-6 [2]	4.78	633.44 → 300.93	55	27
		→ 275.28		27
Ellagic acid	5.56	301.04 → 283.88	60	28
		→ 144.88		36
Rutin	5.70	609.38 → 300.20	60	34
		→ 271.00		60
Quercetin-3-*O*-galactoside	6.07	463.19 → 300.27	57	22
		→ 270.95		44
Quercetin-3-*O*-glucoside	6.42	463.19 → 300.27	57	22
		→ 270.95		44
Quercetin-3-*O*-hexoside	7.13	463.19 → 300.27	57	22
		→ 270.95		44
Kaemprferol-3-*O*-rutinoside	7.23	593.19 → 284.77	60	34
		→ 254.98		60
Quercetin	8.16	301.27 → 150.94	47	20
		→ 120.95		32

[1] The first MRM transition (*m*/*z*) constitutes the quantifier and the second the qualifier. [2] Sanguiin H-6 A was quantified using the transitions of Galloyl-HHPD glucose isomer, which is a dominant fragment of the compound.

Disclaimer/Publisher's Note: The statements, opinions and data contained in all publications are solely those of the individual author(s) and contributor(s) and not of MDPI and/or the editor(s). MDPI and/or the editor(s) disclaim responsibility for any injury to people or property resulting from any ideas, methods, instructions or products referred to in the content.
